# A Real-Time Multiplexed Microbial Growth Intervalometer for Capturing High-Resolution Growth Curves

**DOI:** 10.3389/fmicb.2019.01135

**Published:** 2019-06-05

**Authors:** David C. Vuono, Bruce Lipp, Carl Staub, Evan Loney, Zoë R. Harrold, Joseph J. Grzymski

**Affiliations:** ^1^ Division of Earth and Ecosystem Sciences, Desert Research Institute, Reno, NV, United States; ^2^ Lumenautix, LLC, Reno, NV, United States

**Keywords:** anaerobic growth, aerobic growth, batch culture, growth curves, growth rates

## Abstract

Batch cultures are a low maintenance and routine culturing method in microbiology. Automated tools that measure growth curves from microorganisms grown in traditional laboratory glassware, such as Balch-type tubes, are not commercially available. Here, we present a new MicrobiAl Growth Intervalometer (MAGI) that measures optical density as it correlates to microbial growth by utilizing photo-conduction as opposed to photo-attenuation used by traditional OD measurement equipment. Photo-attenuation occurs when biomass in suspension within a medium blocks and/or diffuses light directed at the detector, such that an increase in biomass results in a decrease in the measured signal. Photo-conduction differs in which the biomass contained in a medium conducts light from the emitter to the detector, where an increase in the biomass results in a corresponding increase in the measured signal. MAGI features software-driven automation that provides investigators with a highly sensitive, low-background noise growth measurement instrument with added capabilities for remote visualization and data acquisition. It is a low maintenance, cost effective, versatile, and robust platform for aerobic/anaerobic cultivation. We demonstrate the versatility of this device by obtaining growth curves from two common laboratory organisms *Escherichia coli* K-12 and *Bacillus subtilis*. We show that growth rates and generation times in *E. coli* K-12 are reproducible to previously published results and that morphological changes of *B. subtilis* during growth can be detected as a change in the slope of the growth curve, which is a function of the effects of cell size on photo-conduction through the medium. We also test MAGI to capture growth curves from an environmental organism, *Intrasporangium calvum* C5, under various media compositions. Our results demonstrate that the MAGI platform accurately measures growth curves in media under various redox conditions (aerobic, microaerobic, and anaerobic), complex and minimal medias, and resolving diauxic growth curves when *I. calvum* is grown on a disaccharide. Lastly, we demonstrate that the device can resolve growth curves for μM concentrations of resources that yield low biomass. This research advances the tools available to microbiologists aiming to monitor growth curves in a variety of disciplines, such as environmental microbiology, clinical microbiology, and food sciences.

## Introduction

Microbial growth and physiology investigations rely on two cultivation techniques: batch cultivation and continuous cultivation, such as the chemostat. These methods produce cells as a function of specific growth rate (μ, h^−1^), and both batch and continuous culture techniques are complementary tools in a microbiologist’s toolbox for investigating the influence of μ on microbial physiology and *vice versa* (Egli, 2015). Batch culturing (Monod, 1949) allows investigators to prepare media of various nutrient compositions for observing all growth phases (lag, exponential, stationary, and death) and to determine the effects of nutrient composition on growth rate in parallel (Ingraham et al., 1983). For continuous culturing (Monod, 1950), μ is set *a priori* by the investigator through a predefined dilution rate of fresh incoming media into the culturing vessel with the cells held in a steady-state of nutrient limitation. This state of nutrient limitation is equivalent to the cusp of the growth curve at late-exponential/early-stationary phase for microorganisms growing in the same media in batch cultures that have nearly exhausted the same growth limiting nutrient (Saldanha et al., 2004). The chemostat allows investigators to focus and study microbial physiology continuously at a single-growth phase (late-exponential/early-stationary) and growth rate.

Both culturing techniques are routinely used for aerobic and anaerobic microbiology, but batch systems are better suited for screening cells for growth under a multitude of nutrient compositions and environmental conditions simultaneously. Many high-throughput technologies are available to screen cells for growth under aerobic conditions, such as plate readers; however, these screening tests are performed in small volumes (μl scale) making most omic-based investigations on the same biological material unfeasible. Furthermore, there are no commercially available high-throughput and automated technologies for screening batch cultures in standard laboratory test tubes in parallel, and many do-it-yourself (DIY)-based systems (Abrevaya et al., 2013; Takahashi et al., 2015; Maia et al., 2016; Sasidharan et al., 2018) require investigators to allocate valuable time to equipment construction and troubleshooting rather than research. Many of these systems are also not easily scalable to accommodate increased parallel experimental replication. Crimp-sealed culture tubes, such as Balch tubes, are routinely used in anaerobic microbiology for culturing microorganisms in larger volumes (e.g., 10–20 ml). However, generating growth curves from these culture vessels requires manual measurements using optical density (OD) or gas production measurements taken at regular intervals (Bräuer et al., 2006), and these measurements can be laborious and time consuming for high-replication experiments, especially with slow growing organisms.

To fill this technological void, we developed an automated high-resolution MicrobiAl Growth Intervalometer (i.e., capturing optical density data at predefined intervals; MAGI) capable of multiplexing biological replicates in standard laboratory glassware. Traditional OD spectrophotometers operate on the principle of photo-attenuation where a light source of specific wavelength(s) passes through an optically transparent vessel and its contained medium to a photodetector that is in direct optical alignment with the light source (Matlock et al., 2011). The amount of light reaching the photodetector is then attenuated (i.e., diffused, absorbed, blocked) by the suspended biomass in the media, and the signal at the detector is proportionally reduced. An inverse function is then applied to the data, so the investigator can observe the growth curve in the traditional shape (lag, log, stationary, and death). MAGI differs from traditional OD spectrophotometers in which growth monitoring is based on photo-conduction, where the light from the emitter that arrives at the photodetector is conducted by the biomass in the medium as a function of surface reflection and/or trans-illumination. As a result, an increase in biomass results in a proportional increase in the detector signal. Fiber optic cables, for example, also operate on the principle photo-conduction where the glass fiber core acts as the photo-conductive medium.

To implement photo-conduction, a photoemitter of specified wavelength(s) illuminates the test tube near its base, and the photodetector is offset from the emitter at a distance sufficient both to increase the optical path and to minimize the direct impingement of light from the source to the photodetector. The benefit of increasing the optical path is a significantly improved analytical resolution; photo-conduction also produces non-inverted data sets.

**Figure 1:**
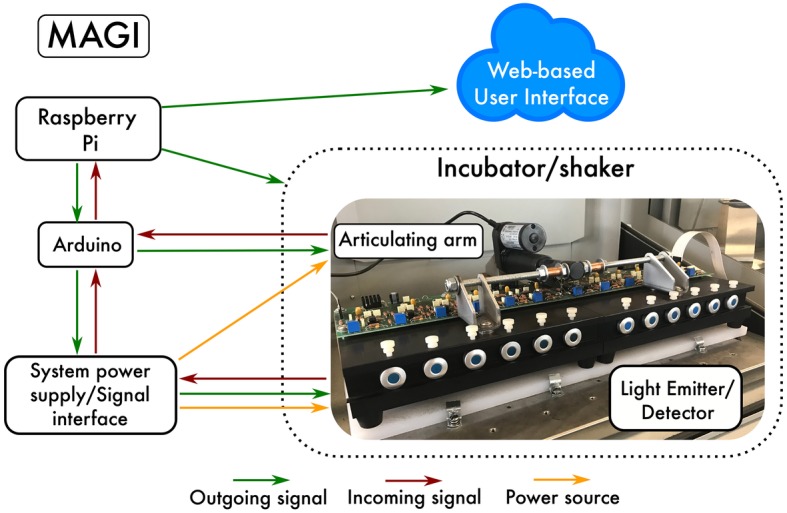
Schematic of MAGI. The Raspberry Pi is a $35 USD computer that controls all of the hardware devices needed to collect growth data from microorganisms. Collected data are configured to be visualized and acquired remotely through a Web-based user interface.

MAGI is compatible with traditional Balch tubes. Standard preparation techniques, such as Hungate technique, are used to dispense media into the culture tubes. Once the vials are sealed with butyl-rubber stoppers and crimped, the investigator can inoculate the various media with the target organism, insert the vials into the MAGI device, and autonomously measure the accurate low-noise growth curves in real time. MAGI uses software-driven automation to operate and collect OD data that are hosted on a server and can be visualized and acquired remotely. The MAGI optical assembly is constructed around a robust fixture containing a light emitter and driver printed circuit (PC) board, detector, and associated trans-impedance amplifier and power regulator PC board. The optical fixture assembly is designed to be contained in the temperature controlled experimental environment, such as an incubator, to allow all active and passive temperature dependent components to reach thermal equilibrium within the experimental environment. An external PC board includes the system power supply and signal interface (Figure 1). The MAGI system support electronics and associated analog-to-digital converter (ADC) and input/output (I/O) components are inexpensive, open platform, robust, and commercially available (Arduino microcontrollers, Raspberry Pi computer). This configuration enables cost-effective construction that can be passed on to consumers to deliver analytical capabilities that are currently unavailable. The device implements 12 parallel culture tube slots, with capacity for expansion constrained only by the size of the incubator/shaker/chamber available to the investigator. The system uses open-source computer languages written in Python, bash, and Arduino (C++) to operate MAGI. Data capture intervals are set through a user-defined function.

## Materials and Methods

*Strain information*: For growth experiments, we used the Invitrogen OneShotTOP10 Electrocomp *Escherichia coli* K12 strain, *Bacillus subtilis* (Harrold et al., 2011), and *Intrasporangium calvum* strain C5. *Intrasporangium calvum* was isolated from a nitrate contaminated groundwater well at the Oak Ridge National Laboratory, Oak Ridge, TN, USA (Lat: 35.97990, Long: 84.27059) as reported by Vuono et al. (2019).

*Media preparation and cultivation*: Lactate/nitrate media for *Intrasporangium calvum* were prepared in a 2 L Widdel flask. After autoclaving, the media were immediately put under an anoxic headspace (N2/CO2 80:20 mix) and sterile filtered (0.2 μm) trace elements, trace vitamins, and reducing agent were added. The media were cooled under an anoxic headspace and buffered with bicarbonate to maintain a pH of 7.2. Hungate technique was used to dispense media into standard Balch tubes (18×150-mm glass tube) (20 ml) pre-flushed with a sterile stream of ultra-high purity (UHP) N_2_ and sealed with blue 1″ butyl rubber stoppers. All cultures were grown at 30°C and shaken at 250 rpm. Nitrate reducing minimal media were prepared with the following final concentrations: NaCl (0.06 mM), NH_4_Cl (1.4 mM), MgCl_2_ (0.2 mM), CaCl_2_ (0.04 mM), KCl (0.1 mM), K_2_HPO_4_ (1.1 mM), NaHCO_3_^−^ (30 mM), cysteine (1 mM) as reducing agent, resazurin as redox indicator, and trace elements and trace vitamin solutions as reported (Yoon et al., 2013; Hillesland et al., 2014). 1 M sterile filtered (0.2 μm) concentrated stocks of 60% w/w sodium DL-lactate solution (Sigma-Aldrich, St. Louis, MO, USA) were diluted into media prior to autoclaving. Culture tubes were inoculated with *I. calvum* cells harvested from a previously grown batch culture in late exponential phase in order to ensure that each experiment began with cells in the same metabolic state. The *I. calvum* cultures were grown at 30°C and shaken at 225 rpm. Microaerobic conditions were achieved by aerobically transferring anaerobic cultures to new anaerobic culture tubes using a syringe and injection needle that was exposed to the air. This was verified by visualizing the media turning slightly pink due to the oxidation of the redox indicator resazurin. Anaerobic transfers were achieved by gassing the injection needle with sterile N_2_ prior to inoculation. *E. coli* was grown in standard Luria-Bertani (LB) broth in Balch tubes that were prepared aerobically, at 37°C, and shaken at 225 rpm. *B. subtilis* was grown in 0.3% w/v Trypticase Soy Broth (TSB; Harrold et al., 2011) in aerobically prepared Balch tubes, incubated at 32°C, and shaken at 225 rpm. Cell staining was performed by making smears of *B. subtilis* suspensions at various growth stages on ethanol cleaned glass slides. The smears were heat fixed and stained with malachite green and safranin, as described by Harrold et al. (2011).

*Data processing and operational software*: The MAGI electrical signal output is analog and is converted to digital by a 10-bit analog-to-digital converter (ADC). Because spectrophotometric measurements are relative, they must be constrained to a quantitative measure by the investigator through standard curves such as cell count. Cell numbers could easily be calculated directly from the ADC units, as reported elsewhere (Vuono et al., 2019). However, to demonstrate the functionality and accuracy of MAGI, we chose to convert our data to OD_600_, which is commonly used by microbiologist to measure growth rates. Data conversion from ADC units to OD_600_ was achieved by collecting a starting and ending OD_600_ value for each culture tube (Hach DR2500 Spectrophotometer). The system was allowed to reach thermal equilibrium within the incubator temperature set-point, and blanks, specific to each medium, were used to adjust the instrument to a relative zero prior to collect the starting data value. Using the values recorded by MAGI, OD_600_ values were then populated using linear interpolation for low-density cultures (<0.6 OD_600_). The detector on MAGI was set to reach saturation when OD_600_ = ~0.6 absorbance, in order to capture low growth yield cultures. Cultures that grew to higher cell densities (>0.6 OD_600_; i.e., *E. coli* aerobically on LB), were sampled manually for OD_600_ at ~30 min intervals on a Hach DR2500 Spectrophotometer to properly convert ADC to OD_600_. The maximum ADC values prior to detector saturation were used as the final values for interpolation to OD_600_. Growth rates were calculated using the package Growthcurver (Sprouffske and Wagner, 2016) in the R environment for statistical computing. The software used to operate MAGI is open source and available on Github^1^.

## Results and Discussion

*Operation*: MAGI is a collection of hardware devices consisting of an Arduino Mega 2,560 microcontroller with an ethernet shield, a Raspberry Pi computer running Linux Ubuntu, an articulating arm (powered by a linear actuator for raising the culture tubes to a vertical position), a power source with analog-based signal amplification for each measurement channel, and a multiplexed light emitter/detector that houses the test tubes (Figure 1). In simplest terms, MAGI runs software to interact with hardware at predefined intervals (i.e., intervalometer). An intervalometer is a device that counts intervals of time. Such devices are commonly used to signal, in accurate time intervals, the operation of some other device. For instance, an intervalometer might activate a device every 30 s. These intervals are triggered by Python scripts stored on a Raspberry Pi computer that communicates with both the orbital shaker/incubator and the articulating arm. The timing of the sampling interval is defined by the user through the software utility cron, which is a time-based job scheduler in Unix-like computer operating systems that are suitable for scheduling repetitive tasks. Cron is driven by a crontab (cron table) configuration file that specifies shell commands to run periodically on a given schedule. Once the sampling interval is defined, MAGI first communicates with the orbital shaker through ASCII commands sent *via* RS-232 serial commands. RS-232 serial port is common to all orbital shaker manufacturers, and while command syntax varies between manufacturers, the command syntax is readily available in user manuals provided by manufacturers.

MAGI executes five operational steps at operator programmed intervals:

1. Communication through software on the Raspberry Pi signals a stop command to the orbital shaker (used to incubate and mix microbial cultures) through a standard serial communication channel.
2. When orbital motion has stopped, a command from the Arduino sends a command to a linear actuator to raise the emitter/detector fixture from its resting position to orient the tubes vertically.
3. After a user-programmed delay to allow bubbles to rise to the surface of the medium, optical density data are collected sequentially from each active culture tube station and recorded.
4. A command from the Arduino reverses the linear actuator lowering the emitter/detector to its resting position.
5. The Raspberry Pi sends a start command through serial communication channel to resume orbital shaker operation. This sequence repeats until the investigator terminates the experiment.

Each data series is time-stamped and populated in tab-delimited format on the Raspberry Pi. Data from each successive sampling interval are appended to the text file until the experiment is terminated. The contents of the data file are displayed in graphical format on a web page hosted on the Raspberry Pi. The web page is coded in HTML and Javascript to provide the investigator with an interactive experience. A simple GUI interface allows the investigator to monitor the progress of the experiment or export and save the data for external use. At the end of the experiment, the data file may be cleared in preparation for additional experiments.

*Technical Specifications*: Replication is essential in microbiology because growth and other physiological responses are sensitive to variations in culture media, preparation techniques, and growth conditions (Egli, 2015). It is relatively easy and inexpensive to prepare many replicates using batch cultures. However, obtaining growth curves from batch cultures, especially in large volumes, can be tedious, time consuming, and low resolution and requires the investigator to remove the test tubes from the incubator to take measurements, which can cause temperature shifts in the growth media. Chemostats, on the other hand, are typically expensive ranging from $20 to 30 K where replication in parallel is often not feasible due to cost. Replication from continuous culture is usually performed sequentially through time, or serially, which may lead to dependencies of autocorrelation and hysteresis. For MAGI, we implemented 12 culture tube measurement cells, split between two emitter/detector blocks (Figure 1). Each block measures 24.8/16.8/7.6 cm (length/width/height), with total system dimensions (including articulating arm) of 49.8/33.6/13.3 cm. This design supports reproducibility for experimental and control groups as well as for screening many cultures under a multitude of growth conditions. The insert size for each measurement cell is designed to fit standard Balch-type tubes with dimensions 18 mm × 150 mm.

**Figure 2:**
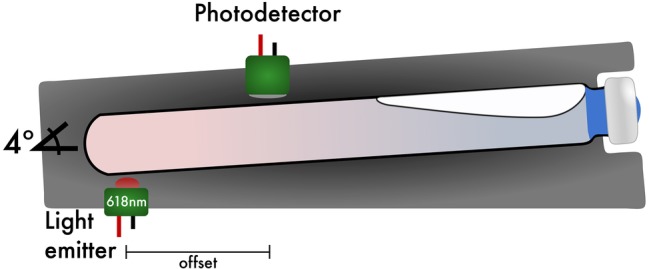
Sagittal view of the light emitter/photodetector and culture tube position. High resolution of MAGI is achieved by off-setting the light emitter from the photodetector. A default tube angle of 4° is implemented to help migrate bubbles produced from biosurfactants toward the top of the culture tube as the instrument stops shaking while still maintaining a horizontal position while shaking.

The light detectors are silicon-based, shielded, and have hermetically sealed quartz windows, which enable wide-band sensitivity. For the light emitter, light emitting diodes (LEDs) of 618 nm were chosen to avoid absorption maxima of resazurin at 600 nm (Reddy and Bordekar, 1999), a common redox indicator used in anaerobic microbiology. The light emitter consists of LEDs that have a nominal dominate wavelength of 618 nm with ±11.5 nm full width at half maximum (FWHM), a peak wavelength of 624 nm, ±30° angle of half intensity, and 90% of total flux is captured within 75° creating a diffused light source. Each emitter is driven by 65 mW of constant regulated energy. Traditional spectrophotometers typically operate through photo-attenuation of collimated light, where the medium being investigated is positioned between an optically aligned emitter and detector (Koch, 1970). In MAGI, the emitter is positioned at the base of the culture tube and is intentionally offset from the detector (Figure 2). This offset increases the optical path length the light must travel, thereby allowing the microorganisms to function as a photo-conductive medium where the microorganisms reflect and/or trans-conduct the light, increasing the analytical resolution of the solution’s optical density. Because of this offset, a minimum of 12 ml of media per tube is required to obtain growth measurements with the configuration being presented here. An offset configuration requires significantly more signal amplification, and so to facilitate photo-conductive operation in MAGI, the photodetector section is comprised of a broad-band silicon photodiode amplifier pair operating in transimpedance mode with an additional low-noise ultra-linear amplifier stage, which is gain tunable by the investigator. As is typical in most interfaced optical density devices, the analog signal output is converted to a digital unit by an ADC. The ADC units can then be readily converted to OD_600_ by recording the starting and ending measured optical density of the culture tube and interpolating the OD_600_ values based on recorded ADC values.

**Figure 3:**
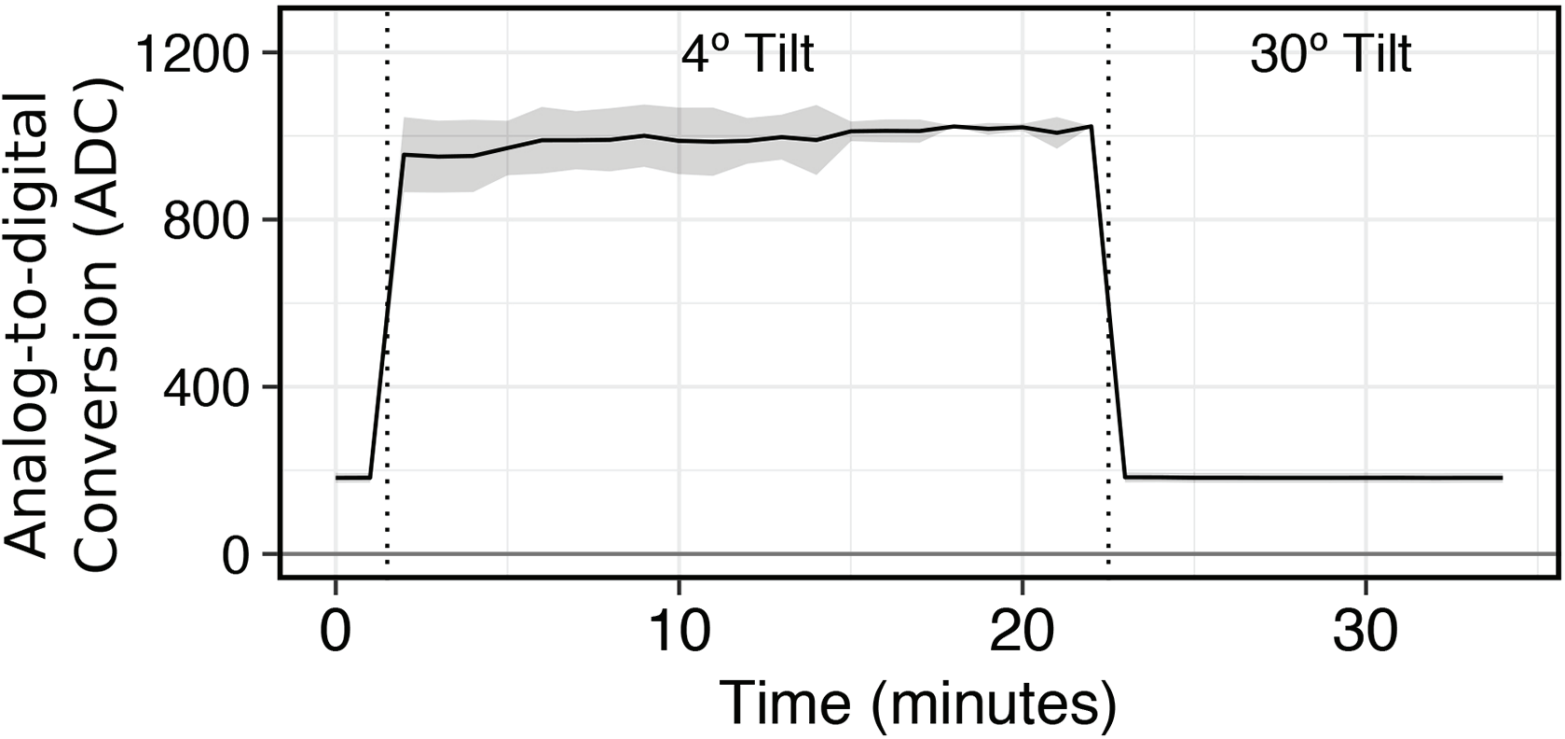
Effectiveness of an articulating arm to orient tube position to a 30° tilt during data acquisition to obviate biosurfactant obstruction of the photodetector.

*Countering Optical Interference from Foaming and Biosurfactant Production*: To validate the need for control from biosurfactant interference on optical readings, we simulated bubble formation in culture tubes (*n* = 12) using standard laboratory soap (0.1% v/v Liquinox). Interference of the optical path from surfactants was tested at 1-min sampling intervals under two states where readings were taken with (1) culture tubes kept at the default horizontal position between and during optical readings (4° Tilt) and (2) with culture tubes lifted by an articulating arm to a 30° angle relative to horizontal during optical readings (30° Tilt; Figure 3). Initially, the first two readings were taken with the tubes oriented at a 30° tilt to define a baseline of 182.1 ± 11.2 ADC units. In between the 1- and 2-min readings, the lead was removed from a relay on the Arduino to prevent activation of the articulating arm. This allowed for optical readings to still be taken but at the horizontal position (4° tilt) where the optical path to the light detectors would be occluded by bubbles at the liquid-air interface. Upon the first reading, the optical signal increased immediately to 955.2 ± 90 ADC units and remained elevated for the duration of position, with an average of 994.1 ± 53.4 ADC units. After the last reading with a 4° tilt (22 min), the lead was reconnected to the relay on the Arduino to engage the articulating arm. This resulted in the optical signal dropping back down to baseline for the remainder of the experiment, with an average of 182.2 ± 11.5 ADC units. These results demonstrate the importance of controlling for biosurfactant interference to prevent aberrant signals.

**Figure 4:**
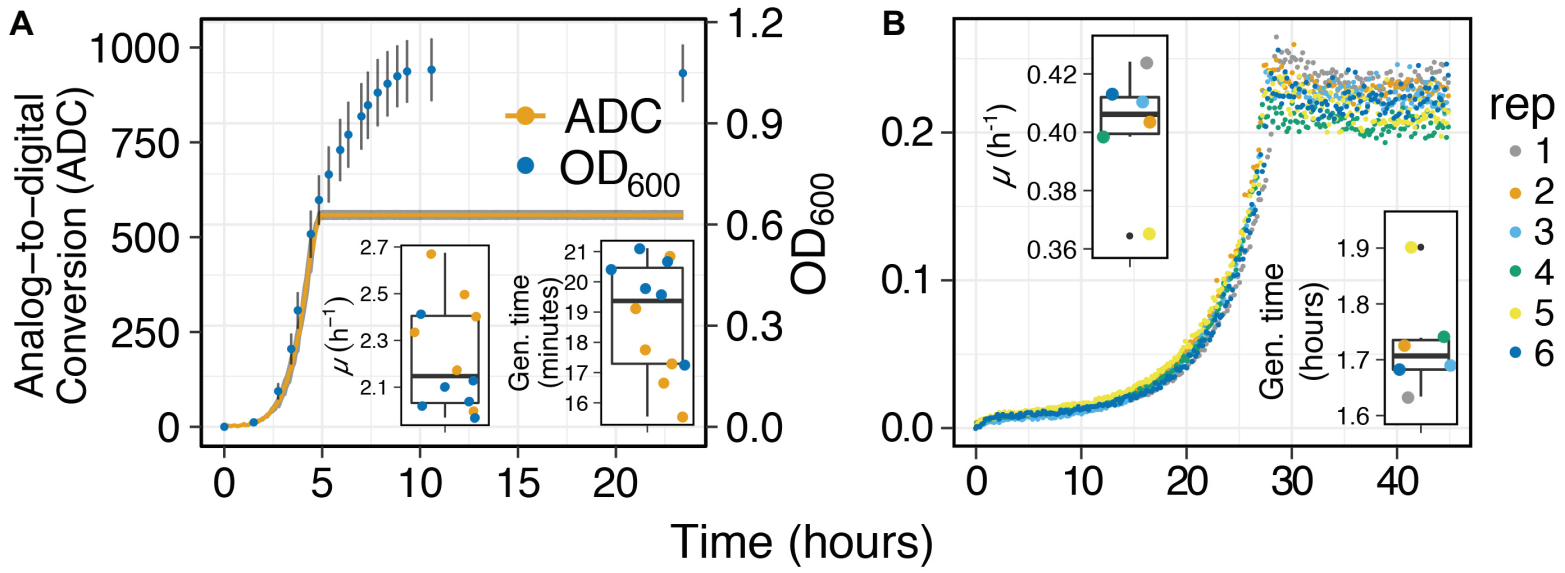
Growth curves of **(A)** *E. coli* grown in aerobically prepared LB media at 37°C and **(B)** *I. calvum* in aerobically prepared minimal media (8 mM Lactate), data adapted from Vuono et al. (2019). Inset graphs are of growth rates (top left) and generation times (bottom right) for each respective sample replicate.

*Capturing Growth Curves from Laboratory and Environmental Organisms*: We selected *E. coli* K-12 to test the effectiveness, accuracy, and resolution of MAGI at measuring batch growth of laboratory organisms. Sealed Balch-tubes enabled us to capture growth curves from aerobic *E. coli* cultures with horizontal shaking. The sampling interval on MAGI was set to 5 min in order to capture the exponential growth phase of *E. coli*. We also collected manual exponential phase OD_600_ measurements of the same culture tubes taken at ~30-min intervals to compare MAGI results with traditional technologies. Results show a typical *E. coli* growth curve with MAGI measurements and manual OD_600_ measurements tracking in sync from lag phase to exponential phase (Figure 4A). The gain on MAGI was adjusted to enable resolution of biomass up to an OD_600_ of ~0.6 absorbance. After an OD_600_ of ~0.6, the MAGI curve reached saturation due to reaching the maximum resolution of the light detector under the instrument settings. Reaching saturation did not, however, prevent standard microbial growth parameters to be calculated from MAGI. The specific growth rates (μ) of MAGI and OD_600_-obtained growth curves were 2.34 ± 0.2 h^−1^ and 2.11 ± 0.16 h^−1^, respectively, with a generation time of 17.8 ± 1.8 min and 19.8 ± 1.4 min, respectively (Figure 4A Inset). These parameters were not significantly different between instruments (*p* = 0.08 and *p* = 0.08, for μ and generation time, respectively; two-tailed *t* test) and are congruent with values reported elsewhere under similar growth conditions (Berney et al., 2006). These results demonstrate the ability of MAGI to accurately measure growth curves where standard microbial growth parameters can be calculated.

We next generated growth curves from an environmental organism, *I. calvum*, grown with 8 mM lactate as carbon source and electron donor and 3 ml of sterile air injected into the anaerobically prepared vial. *I. calvum* is an Actinobacterium of the family *Intrasporangiaceae* (del Rio et al., 2010). It known for producing branched mycelium with intercalary sporangia in the mycelial hyphae (Kalakoutskii et al., 1967), the function of which is unresolved (Lechevalier and Lechevalier, 1969). *I. calvum* has been isolated from air (Kalakoutskii et al., 1967), terrestrial subsurface (Green et al., 2010; Vuono et al., 2019), has been found in activated sludge bioreactors (Vuono et al., 2016), and is known for its ability to dissimilate nitrite *via* a dual pathway of denitrification and respiratory ammonification (Vuono et al., 2019).

When *I. calvum* was grown with lactate and O_2_, we observed growth curves where all replicates were in sync during each growth phase (Figure 4B). *I. calvum* displayed an abrupt transition to stationary phase, which was likely due to the depletion of O_2_ in the culture vessel. From these growth curves, we were able to calculate a specific growth rate of 0.4 ± 0.02 h^−1^ and a generation time of 1.73 ± 0.09 h (Figure 4B Inset).

**Figure 5:**
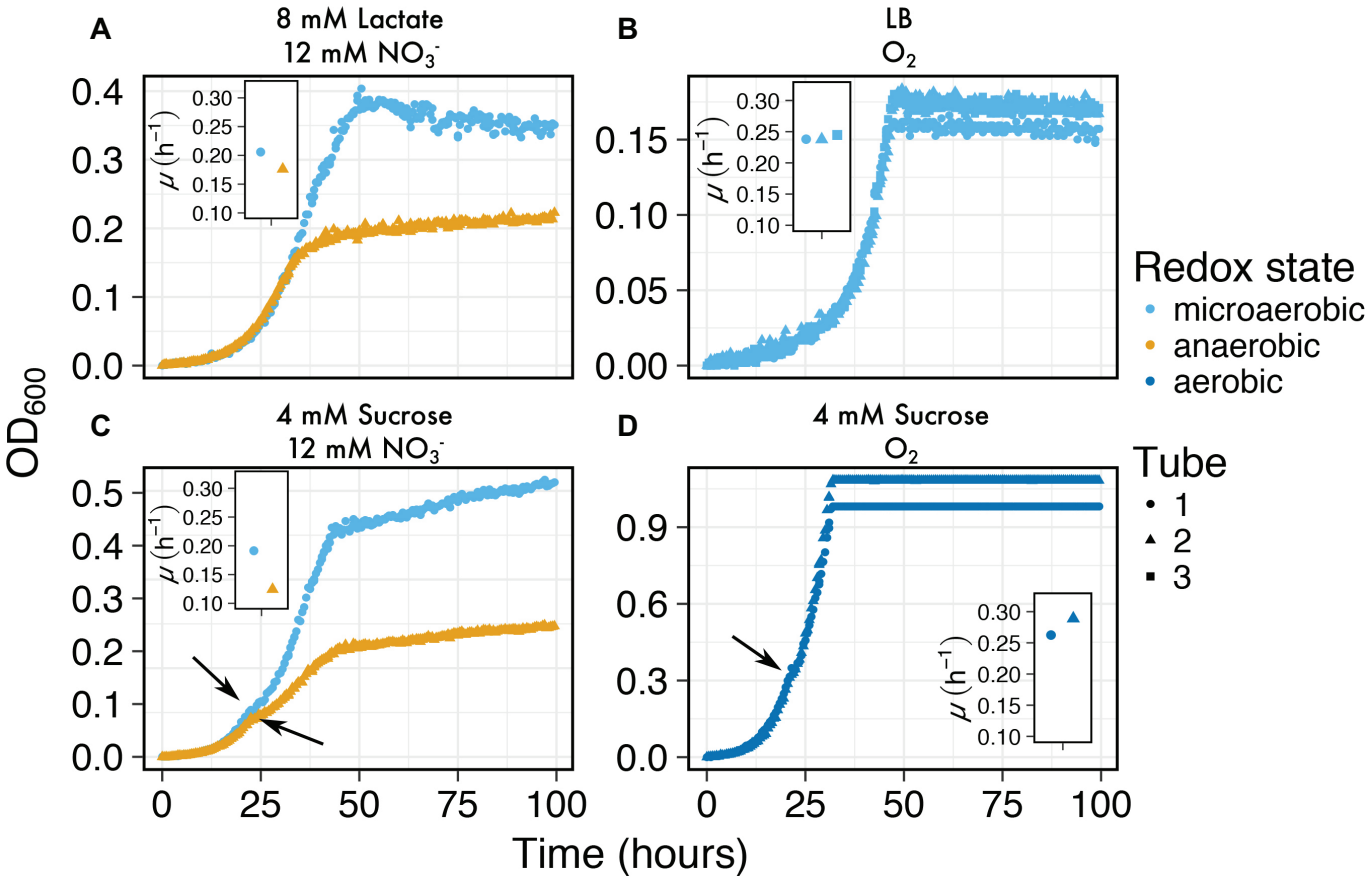
Growth curves of *I. calvum* at various media compositions at 30°C: **(A)** microaerobic (light blue circles) versus anaerobic (orange triangles), **(B)** complex media (LB), and growth with a disaccharide under **(C)** microaerobic (light blue circles) versus anaerobic (orange triangles), and **(D)** aerobically prepared media. Arrows in **(C)** and **(D)** indicate shoulders of the growth curve where diauxic growth transition occurs. Inset graphs are of growth rates for each respective sample tube.

Next, we explored whether we could use MAGI to screen a variety of media compositions with *I. calvum* cultures in order to resolve minor differences in growth depending on variations in starting conditions. This type of screening can be used to further pursue more rigorous lines of research that may be significant to the experimenter. The media compositions tested were microaerobic versus anaerobic (Figure 5A), complex media (Figure 5B), and growth with a disaccharide in order to capture diauxic growth (Figures 5C,D). These results showed that MAGI was able to capture independent growth curves with sufficient resolution from anaerobic versus microaerobic conditions, which were also found to have slightly different growth rates (Figure 5A; microaerobic *μ* = 0.2 h^−1^; anaerobic *μ* = 0.18 h^−1^). The cultures growing in complex media (LB) under microaerobic conditions exhibited highly reproducible growth rates (*μ* = 0.24 ± 0.004 h^−1^) indicating the accuracy of MAGI to resolve growth curves in complex media (Figure 5B). When *I. calvum* was grown on the disaccharide sucrose, we tested whether the sampling resolution of MAGI could capture growth variation during the rapidly changing biomass phase of exponential growth. For the cultures grown under microaerobic and anaerobic handling (Figure 5C), MAGI was able to resolve the characteristic shoulder (short lag period) of diauxic growth (Monod, 1949) at approximately 20 h. These curves also had different growth rates during the second exponential phase for each culture tube (Figure 5C; microaerobic *μ* = 0.19 h^−1^; anaerobic *μ* = 0.12 h^−1^). Similar results were observed under aerobic growth conditions (Figure 5D). Overall these results show that MAGI’s multiplexed configuration with high-resolution data capture is useful for screening multiple growth conditions in parallel, which could be further pursued by the experimenter.

**Figure 6:**
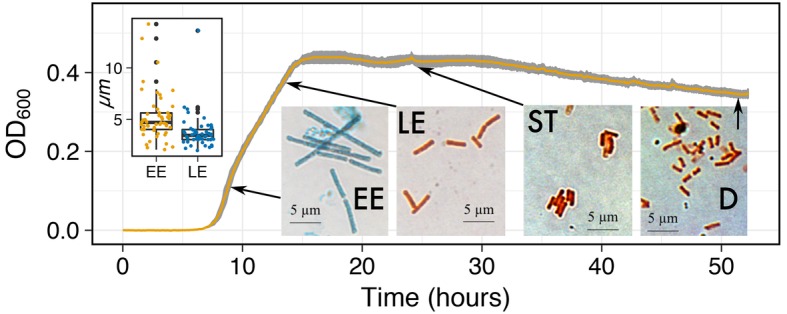
Growth curves of *B. subtilis* on 0.3% TSB at 32°C. Inset micrographs are shown at their respective growth phases (EE, Early Exponential; LE, Late Exponential; S, Stationary; and D, Death) to identify the effects of morphological changes on the growth curve. Inset boxplot shows the difference in cell length (μm) between the longer rod/filament-like morphology during EE and the traditional rod-shaped morphology during LE.

*Capturing morphological changes during growth*: Microbial cells are found in a variety of sizes and morphology, which all have different photo-conductive properties. Microbial cells are also known to change size and morphology during growth due to different resource constraints (Egli, 1991) and life-history strategies (Logan and De Vos, 2011), such as spore formers. To test MAGI’s ability to detect morphological shifts of the spore forming *Bacillus subtilis*, we grew the bacterium in 0.3% TSB (Figure 6), a media composition previously reported to promote spore formation (Harrold et al., 2011). The cultures began exponential growth at ~7 h. At ~9 h, we observed a change in the slope of the exponential growth curve. Afterward, we observed a typical transition to stationary phase (S) and death (D). Prepared smears of *B. subtilis* cells stained with malachite green and safranin were observed with light microscopy for samples collected before and after the 9-h time point to investigate the change in growth rate slope. The change in the slope of the growth curve coincided with a morphological shift from rod/filament-like cells (5.25 μm ± 2.36) during early exponential (EE) to the traditional rod-shaped morphology (3.74 μm ± 1.66) at late exponential (LE) (Inset boxplots and micrographs). The increased cell size during EE was significantly larger than the cells in LE (*p* < 0.001; one-tail *t* test) and also appeared to retain the malachite green stain in the cell wall over the safranin red stain. To our knowledge, evidence for this shift in *B. subtilis* vegetative cell morphology has not been published. The results of a shift in the slope of the exponential growth curve highlight the sensitivity of MAGI to characterize putative changes in cell morphologies that affect the transmittance of light through the medium. More work is required to understand how sporulation and other morphological changes affect photo-conductance and the MAGI signal.

**Figure 7:**
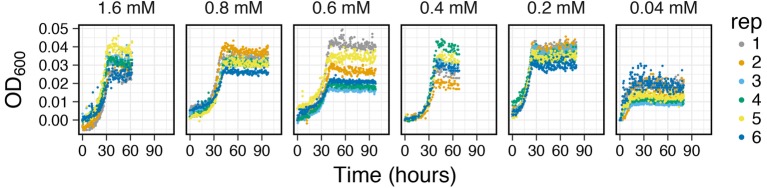
Growth curves of *I. calvum* at various carbon concentrations indicating the sensitivity of MAGI to detect growth curves of cells at low growth yields. Data have been adapted from Vuono et al. (2019).

*Capturing Growth Curves from Low Resource Concentration Mediums*: Growth yields from anaerobic metabolisms are often times very low due to the lower free energy yields (∆G°) of electron donors and acceptors used in these reactions (Thauer et al., 1977). Furthermore, measuring growth curves from environmentally relevant resource concentrations (μM amounts) is also difficult due to the detection limits on most OD detection devices. The analog-based signal amplification on MAGI allows users to adjust the detection resolution based on anticipated growth yields. To test this feature on MAGI, we prepared anaerobic media of *I. calvum* cultures with a range of carbon concentrations that varied from mM to μM ranges (Figure 7). Results show that with decreasing carbon concentration, we were able to resolve growth curves with optical densities as low as 0.008 OD_600_ (0.04 mM lactate concentration, replicate 3) with a group average of 0.015 ± 0.004 OD_600_. These results demonstrate the capability of MAGI to resolve growth curves from media with low substrate concentrations and low biomass yields. Furthermore, in a previous study, we were able to resolve higher growth rates among the treatments (at low carbon concentrations), which selected for the ammonification respiratory pathway over denitrification in *I. calvum* C5 (Vuono et al., 2019).

## Conclusion

Batch cultures are a low maintenance and routine culturing method in microbiology. Automated tools that measure growth curves from anaerobic microbial cultures grown in traditional laboratory glassware, such as Balch-type tubes, are lacking. Here, we present a new microbial growth intervalometer (MAGI) that uses software-driven automation, remote visualization, and data acquisition. We demonstrate the utility of this device by first showing that growth rates and generation times in *E. coli* K-12 are reproducible to previously published results. We then tested the ability for MAGI to measure growth curves of an environmental organism, *I. calvum*, under various media compositions. Our results demonstrate that MAGI is a versatile platform for microbiology research.

## Author Contributions

DV, BL, CS, ZH, and JG wrote the manuscript. CS designed and constructed the instrument. BL designed and built the software to run the instrument. DV, EL, and JG contributed to the conception and design of the study. DV, EL, and ZH performed the lab work. DV, EL, ZH, and JG analyzed the data and interpreted results. All authors contributed to manuscript revision, read, and approved the submitted version.

### Conflict of Interest Statement

Lumenautix LLC funded the R&D of the instrument and holds the intellectual property, author CS is the Lumenautix CEO. Lumenautix had no role further role in funding the study.

The remaining authors declare that the research was conducted in the absence of any commercial or financial relationships that could be construed as a potential conflict of interest.

## Footnotes

^1^https://github.com/dvuono/MAGI
